# Community perspectives on persistent transmission of lymphatic filariasis in three hotspot districts in Ghana after 15 rounds of mass drug administration: a qualitative assessment

**DOI:** 10.1186/s12889-018-5157-7

**Published:** 2018-02-13

**Authors:** Collins S. K. Ahorlu, Eric Koka, Susan Adu-Amankwah, Joseph Otchere, Dziedzom Komi de Souza

**Affiliations:** 1 Department of Epidemiology, Noguchi Memorial Institute for Medical Research, University of Ghana, P.O Box LG 581, Legon-Accra, Ghana; 2 Department of Sociology and Anthropology, University of Cape Coast, Cape Coast, Ghana; 3 Department of Parasitology, Noguchi Memorial Institute for Medical Research, University of Ghana, P.O Box LG 581, Legon-Accra, Ghana

**Keywords:** Lymphatic Filariasis, Drug fatigue, Key-informants, MDA, Community perspectives, Qualitative, Ghana

## Abstract

**Background:**

The Global Program for the Elimination of Lymphatic Filariasis (GPELF) started operation in 2000 and aimed at eliminating the disease by the year 2020, following 5–6 rounds of effective annual Mass Drug Administration (MDA). The MDA programme took off in Ghana in 2001 and has interrupted transmission in many areas while it has persisted in some areas after 10 or more rounds of MDA. This study was to appreciate community members’ perspectives on MDA after over 15 years of implementation. Findings will inform strategies to mobilise community members to participate fully in MDA to enhance the disease elimination process.

**Methods:**

This was a qualitative study, employing key-informant in-depth-interviews. Respondents were selected based on their recognition by community members as opinion leaders and persons who were knowledgeable about the topic of interest in the community. A snowball sampling technique was used to select respondents.

**Results:**

Respondents were well informed about the MDA with most of them saying, it has been implemented for over 12 years. They were aware that the MDA was for the treatment/control of LF (elephantiasis). It came to light that MDA compliance was affected by five related barriers. These are; Medication, Personal, Health system, Disease and Social structure related barriers. Adverse effects of the drugs and the fact that many people perceived that they were not susceptibility to the infection have grossly affected the ingestion of the drugs.

**Conclusion:**

There is a need for community mobilization and promotional activities to explain the expected adverse reactions associated with the drugs to the people. Also the importance of why every qualified person in the community must comply with MDA must be emphasized.

## Background

Lymphatic filariasis (LF), which results from infection with the mosquito borne nematode parasite *Wuchereria bancrofti*, is an important public health problem that has been targeted for elimination by 2020. It affects 120 million people in 73 countries where 1. 46 billion are at risk of acquiring the infection through infectious mosquito bites [1]. Lymphatic filariasis is rarely fatal, but its clinical manifestations carry grave debilitating personal and socioeconomic consequences for the infected and the affected [2–4]. The commonest clinical manifestations of LF are acute attacks of adenolymphangitis (ADL), characterised by episodic attacks of fever associated with inflammation of the inguinal lymph nodes, testis, spermatic cord or a combination of these, and disfiguring chronic conditions like lymphoedema/elephantiasis, which may render victims to social scorn and stigmatization in the community and thereby making them lose self-esteem [2, 3, 5].

The development of diagnostic tools and drugs for treating the filarial worms has stimulated the hope that the infection could be eliminated. As a result, the World Health Organization, in collaboration with pharmaceutical companies and endemic country governments, launched the Global Programme to Eliminate LF (GPELF) in 2000. The drugs of choice for the global elimination programme were Ivermectin (IVM) in combination with Diethylcarbamazine (DEC) or Albendazole (ALB) [1]. These drugs have the ability to only clear microfilariae (mf) with little or no macrofilaricidal activity [6]. However, it is assumed that a reduction in the mf load will lead to a reduction in adult worms, which will eventually lead to an interruption in transmission [7–9]. At inception, it was estimated that 5–6 rounds of mass drug administration (MDA) was all that would be required to eliminate the disease. However, Ghana have since organised 15 consecutive annual rounds of MDA in several implementation units without interrupting transmission in some communities, which have been labelled as ‘hotspots’ [10]. It is instructive to note that, in Ghana, MDA coverage rates were more than the required 65%, in 29 sentinel sites, which have persistent residual infections, with microfilariae prevalence rates > 1% [11].

The failure to interrupt LF transmission in some endemic communities in Ghana after 15 years of annual MDA requires that any alternative and effective MDA regimens and strategies to be adopted must incorporate local perceptions and attitudes towards MDA to aid community mobilization for effective delivery. It is therefore imperative to seek answers to questions like: Are people in hotspot communities taking the medicine as expected to interrupt transmission? Are community members comfortable with, and willing to continue ingesting the intervention drugs? Is there any drug ingestion fatigue among the study population? It is hoped that providing answers to these questions from community members’ perspectives will be vital for any revision of the programme implementation to ensure maximum cooperation and participation to enhance the effectiveness of the elimination agenda. This is important in the light of current efforts to test alternative treatment regimens, such as bi-annual treatment schedules is being recommended [1], to facilitate interruption of transmission in the hotspot areas.

### Objectives

The objective of this qualitative study was to determine the community members’ participation and ingestion of the intervention drugs. The study also aimed at generating appropriate information from community perspectives to inform promotional strategies to rekindle participation and promote the ingestion of the intervention drugs in hotspot communities.

## Methods

### Study site

The study was conducted in 18 lymphatic filariasis endemic villages in three districts (Ahanta West, Nzema East and Nzema West Districts) in the Western Region of Ghana. Detailed descriptions of the study area, its population and the endemicity of lymphatic filariasis in the villages, before the commencement of MDA, have been presented previously [12]. Briefly, the study districts are inhabited mainly by the Nzema (Nzema East Municipality and Ellembelle district) and Ahanta (Ahanta West district) speaking people. Ahanta and Nzema are closely related dialects with little variations; both belong to the larger Akan speaking people of Ghana. Farming and fishing are the main occupations of the people in the study districts. However, small scale mining and trading also employed a good number of residents. Ghana’s budding oil industry is located off the shores of these districts. Two districts (Nzema East and Ellembelle) started MDA implementation in 2000 while Ahantan West started in 2002. A survey conducted in 2014 shows LF (ICT) prevalence of between 8.2% and 23.5%.

### Study design

This was a qualitative study, employing key informant in-depth interviews for data collection. The study was carried out in May 2017. The study was undertaken in 18 communities, in the Western region of Ghana, selected for a community-based bi-annual treatment with Ivermectin and Albendazole [13].

In-depth interviews [14] were held with respondents, who were mainly; traditional rulers, opinion leaders and community-based drug distributors, to explore the perceptions and behaviours towards the MDA which has been administered for 15 years in the three districts. The In-depth interviews were conducted using a questionnaire guide administered by a Research Assistant who spoke the local language fluently. The first author (CSKA, a Social Epidemiologist), who also understood the local language, was present as an observer at all the In-depth interview sessions.

In all, four in-depth interviews were held in each community. These involved the chief/his representative, the queen-mother (traditional women’s leader) or her representative, the community-based drug distributor and one other opinion leader identified by the chief and confirmed by the queen-mother. The socio-demographic characteristics of respondents are presented in Table 1. Respondents were made up of 38 men and 34 women aged between 25 and 60 years. We stopped the interview at the fourth person because the third interview contributed very little new information and the fourth interview produced virtually no additional information from the first three interviews. Thus, a theoretical saturation point was reached in each community. None of those requested to participate in the study declined. In each community, in-depth interviews with two key informants were repeated with the same individuals to determine the reliability of responses and there were no major differences between the first and second rounds of responses. Proceedings from in-depth interviews were tape-recorded and notes were taken to complement the recorded audio.

**Table 1:** Socio-demographic Characteristics of Respondents

Respondents	Male	Female	Age (Years)	Total
Chief/his representative	18	0	40–60	18
Queen mother/her representative	0	18	40–60	18
Drug Distributors	10	8	25–55	18
Opinion Leaders	10	8	30–50	18
Total	38	34		72

The dialogues were transcribed directly into English and entered into a computer in a pre-coded format using Microsoft word, which was then imported into the MaxQda software for qualitative data analysis. The MaxQda software was used to further code relevant overlapping segments of the narratives and categorized statements for content analysis to select representative and relevant responses for presentation. There were very little obvious differences in individual responses of participants, however, any such differences identified were presented and discussed to show contrasting opinions and positions.

## Results

Community members in our study area were well informed about the MDA that has been going on in their communities for over 12 years and could describe it vividly. They were also aware that it was meant to treat/control LF or elephantiasis. They were also aware that some people were not taking the medicine consistently, even drug distributors themselves. Thematic analysis showed that five closely related barriers were identified as impeding the ingestion of the drugs and we present the findings under these headings for clarity.

### Medication barriers

It was reported that some people were not taking the medicine (MDA) as a result of the medicine/drug related barriers. These barriers included: the fear of side effects, which were reported to be mostly dizziness, rashes and general weakness. Respondents were of the view that once someone had complained about side effects, others simply did not try taking the drugs to know it for themselves. It came to light that some people collected the drugs and later threw them away at the blind side of the volunteers just to avoid experiencing side effects. The following representative narratives explain this position:

*I remember very well that somewhere in 2008, several children were given the drug and they had serious side effects including rashes, dizziness and swollen faces and legs […], some became very sick. Since then, the news had spread like wildfire, and as a result of that, many people in many communities do not want to take the drug again* (opinion leader).

*They refuse because of the severe side effects that others have experienced after taking the medication* (Chief – Male traditional leader)*.*

*Those who don’t take the medicine think that it triggers other illnesses in addition to other side effects like dizziness and rashes* (Queen mother – Female traditional leader*).*

*I know that pregnant women were not supposed to take the medicine […] Those who usually refuse to take the medicine are those who had experienced some ill health after taking the medicine or hear others say so. They are afraid that perhaps they will not be able to go about their economic activities when they take the medicine* (female Opinion leader).

*Some complain that when they take the medicine it usually brings out other diseases, others say that they were frightened by the severe side effects which sometimes render them unable to go about their daily works […] There are some who think that it may cause other problems for them when they take it* (male Opinion leader).

*I took the drug before and had lots of side effects […] I was itching and had rashes all over my body. […] I stopped taking it. I can say that, I stop taking it because of the side effects; many people do not want to take it too. The worm medicine is good but the side effects put fear in people* (volunteer distributor).

### Personal and disease related barriers

It came to light that some people either did not take the medicine or stop taking it because they don’t like taking medicine of any kind. Others also felt that the medicine had no impact on their health as they could not see any changes in their bodies after taking the medicine. Others refused to participate in the MDA because of the perception that they were not susceptible to LF infection, simply because they have no clinical signs of the disease, especially elephantiasis and hydrocele. Thus, once an individual perceived that she/he is not at risk of LF infection, the motivation for taking the medicine was low. In a similar vein, it was reported that community members know that only very few people among them have the infection. This knowledge was based on the number of clinical presentations of the disease seen in the community. Respondents maintained that people can point out the homes or compounds where those few cases were found and therefore questioned why those compounds were not targeted for treatment instead of the whole community. Some community members also hold the conviction that medicines are meant for treatment/curing illnesses but not to serve as vaccines. This position makes people to hold the view that it should not be taken by people who are well and in no pain. The ‘healthy’ people will reportedly prefer vaccines as means of preventing infections (diseases) to medicines.

*Some people just intentionally refuse to take the drugs […] many people also avoid taking the drugs based on hearsay and perceptions about the side effects of the drug* (Queen mother - female traditional leader).

*Some people refused to take the drug because they think that they are not sick […] even me (a distributor), I stopped taking it not because of side effects but I think that I did not have the disease but now, you have educated me to know that I am at risk like any other person in the community, so I will resume taking it* (volunteer drug distributor).

*Personally, I don’t take the medicine every year because I just don’t like the distributor in this community […]. He likes drinking alcohol too much and sometimes he will be asking for money from people. He is a lazy person and therefore put me off* (Male opinion leader).

*I took the medications for about three times and stopped because the distributors were not telling us why we should continue to take the medicine. […]I do not have Dugba/Gyepim (elephantiasis) or Etow/Etso (hydrocele) so why should I be taking medicines, which rather makes me sick? […] now you (researchers) have explained to us very well why we should continue to take it till the germ is completely killed in everybody’s blood* (male opinion leader).

*Some people think that anytime they take the medicine, they experience other diseases and so once they think that they do not have the disease (filariasis), why should they be taking the medicine, so they stop […] I know that some people who took the medicine for less than 3 time* (volunteer distributor).

*Initially everyone took the drugs, but the side effects were many […] many people was reluctant to take it due to fear of side effects […] Even this morning before you people came here, I heard someone saying that if it was the filariasis drugs that you* (the research team) *were bringing, then she would not take it because she had rashes previously after taking it* (female opinion leader)*.*

### Health system related barriers

Some of the community-based volunteer distributors were not trusted by the people. Some of them were considered as political activists who were trying to make political gain with the programme. Also, respondents were of the view that there was weak supervision from the district health management team making it possible for the distributors to behave like medical doctors (health professionals). It was reported that, some of the volunteers acted as if they were doing the community a favour by distributing the drugs. Some respondents were of the view that, some of the volunteers were not committed to ensuring that people take the medicine. Also, in a few communities, the volunteer distributors were coming from different communities and this, reportedly, affected cooperation from the people. For the people, it was an indication that they were not capable of helping themselves. They maintain that those ‘foreign’ distributors do not know everybody in the community and therefore could not look for those they did not meet at home.

*I think that the time the volunteers come to distribute the drug affects the intake of the drugs […] they come at their own convenience. I think the time should be changed to the evening so that many people can take it […] they (distributors) usually come after community members were gone to their farms or fishing and we cannot say that they should come very early as many people will not have eaten then and therefore be unwilling to take the drugs* (Queen mother - female traditional leader).

*[…] if you want all of us to take the medicine, then the distributors should be allowed to keep the drugs with them for longer time, so that they can have enough time to reach everybody. Sometimes before you realized they have sent the drugs back to Axim […] I think that is also affecting the coverage* (male opinion leader).

The volunteers were asked to share the reasons why they think that some people were not taking the medicine consistently on annual basis and whether in their opinions the mode of distribution was affecting participation. They generally disagreed that the mode of distribution was affecting participation but rather indicated that it had to do with the efficacy of the medicine, some people not being available in the community during MDA, Poor community mobilization and communication on the activities of the distributors. These positions were aptly summed up in the following narratives:

*The problem is not about the mode of distribution. Such people do not have confidence in the efficacy of the drug. Such perceptions are fed into some people and they refuse to take the drug. The time and day of distributing the drug may affect participation but I do not think it is a big problem […]. Those who are willing to participate do make themselves available to take it anyway. We (volunteers) move from house to house and sometimes go back again if most people were not in the house* (volunteer distributor).

*At times, many people are not at home or in the community during the distribution. […] last year, the turnout was very low in this community because there was no local radio to make announcement for people to stay at home […] now, we have a local radio, so I believe that the number will increase since they will be doing announcement* (volunteer distributor).

The distributors, were of the view that, the arrangement where the efficiency of volunteer distributor was measured by the number of pills received and the number returned, opened the door for dishonesty, thus, some volunteers may have marked people who did not take the drugs as having received it, only to throw those drugs away, just to demonstrate that they had performed well. In this regard they recommended strict supervision by the health authorities during MDA. Others thought that the refusal of health facilities (hospital and clinics) to treat those who suffered adverse reactions after taking the medicine was also affecting the level of participation in the MDA programme. These sentiments are presented in the following narratives:

*[…] even me as a distributor know that sometimes, some of us will throw some of the medicine away and then mark people in the register as having received the medicine just to show that we are hardworking […] I think that the health officers from the district office or the hospital/clinics should visit us in the communities to make sure that we do the right thing* (female Volunteer distributor).

*Most of my colleagues think that it is a voluntary work and therefore do not take the work very serious […] When they visit a home the people are there, they just tick the names and throw the medicine away, so that their coverage will be very high. I will not be surprised if you go round with the record form and realise that some people will be ticked for taking the medicine far more than they have actually done […] we should be given more time to distribute the medicine, so that we can take time to find everybody in the community* (male volunteer distributor).

*In my view the hospital is not helping us […] when people take the medicine and become sick, when they go to the hospital they are asked to pay before they are given medicine. You see, the person was not sick till you gave him your medicine, so when he/she become sick after taking your medicine, you have to treat him/her for free […]to ask such a person to pay, I find it difficult to understand* […] (male volunteer distributor).

*I think, there is the need for more education about the possible side effects of the drugs so that people will know about them before taking the drugs […] They should also be made to understand that the side effects are normal, just as you (research team) told us that we all react to medicines differently but that does not mean that the drug is not good for those who react to it* (volunteer distributor).

### Social structural barriers

Structural barriers reported have to do with the social structural limitations imposed mostly by family power and decision-making relationships. It came up that some parents had prevented their children from taking the medication, even when the children were willing to take the medicine. Also, it was reported that some husbands have prevented their wives from taking the medication. Again, some ladies faked pregnancy in order to be exempted from ingesting the MDA drugs.

*I know that some parents have stopped their children from taking the drugs […]. In this community, once your father or husband asked you not to participate in something, you have to obey else there will be family troubles, even the child could be beaten for disobeying the father* (female opinion leader).

*I know of a household where the man had stopped every member of the household from taking the drugs […], the man believes that none of his household members have the worm in their blood […] I don’t even know how he came about that information. […] initially they took the medicine, maybe two or three times, after that anytime I go there with the drugs, none of them will take it and it was when I asked one of the children that she told me that they were told by their father not to take the medicine again* (volunteer drug distributor).

It also came to light that some community leaders felt that they were not involved or consulted in the selection of the volunteer distributors, some of whom have turned themselves into “village doctors” who came into the community to command everybody around and therefore asked to be made to supervise the activities of the distributors. Respondents were of the view that when this is done, they (community/opinion leaders) will not only supervise the volunteers but also help to mobilize community members to take the medicines. They maintain that, as community leaders, they have the authority to legislate and sanction recalcitrant members of the community traditionally, so it was imperative that health officials recognize them first. Some respondents reported that some of the volunteers were doing politics with the programme and therefore naturally excluded some people who did not support the political party of the volunteers. When pressed further regarding what they mean by ‘doing politics’ it was explained that supporters of the two major political parties in Ghana were trying to politicize the programme with each of them claiming that the programme was instituted by their party to show their concern for the people. These positions were aptly represented in the following responses:

*I am the chief of this community but I do not know how the distributors were selected […] some of them were arrogant and very disrespectful […] they come to order all of us about as if they are our doctors. I think that the doctors should have a meeting with us and tell them in our presence that they are under us for us to supervise them* (Chief –traditional leader).

*[…], you know, we chiefs are not supposed to support any political party openly, so we can select volunteers who will be accepted by all but not this partisan boy who does not respect anybody in this community […] he also drinks a lot of alcohol and if we were consulted, he will not have been selected as a volunteer to distribute the drugs [he is always fighting with people* (Chief –traditional leader).

*As for me, I think that the politicisation of the programme by some of the volunteers is the reason why some people are not taking the drugs […] you cannot come to my house and argue on political issues with me because we all support different political parties and if I don’t like your party or what you are saying about my party, I will ask you to leave my house with your drugs* (male opinion leader).

Respondents were asked to freely suggest what can be done to get most people, if not all, to take the medicine and varied responses were provided. The main suggestion was on community mobilization in the forms of sensitization and education on the benefits of the medicine as well as the side effects after taking it. Respondents were of the view that when the people understood the benefits for taking the medicine for many years, they will be willing to tolerate the adverse reactions of the drug. Some also were of the view that when people are assured that they will be treated at the health facility free of charge when they experience adverse effects, they will be willing to take it. Some others want the distribution to be done on more convenient times of the day, in the morning and evening and especially on Sundays where most people are at home.

### Personal experience with the drugs

Respondents were asked if they had taken the drugs before and very interesting responses were provided. It came to light that virtually all respondents had taken the drugs before, except one volunteer distributor. However, most of the respondents had stopped taking the drugs while others were taking it intermittently, having missed some of the rounds. These positions were represented in the representative narratives below:

*I have a brother who had elephantiasis, so I always take the drug every year […] I have never had any side effect after taking the drug, though sometimes I feel some itching and dull, I will not call them side effects, sometimes you can wake up and feel the same without taking any drug […] I have taken it for more than 12 times if I am mistaken [laugh] I even lost count of it* (Chief – traditional leader).

*Yes, I have taken it about 7 times […] I missed out on a number of occasions because I was not at home when it was distributed […]. Well, I don’t think I will stop taking the medicine again because you (research team) have explained it to me properly, I have to help my children, grandchildren and great-grandchildren by taking the drug to uproot the disease from our community* (male opinion leader).

*Yes, I think, I took it for about 5 times […] I did missed out sometimes deliberately to avoid the side effects of the drugs, some of the times the distributor did not meet me at home. I don’t think I will stop taking the drug again because I think it is helping us […] now we have fewer people with elephantiasis in the community. I must confess that when I am sick at the time of sharing the drugs, I may not take it because it will make my sickness worse* (Female opinion leader).

*Yes, I have taken the drugs before. Initially when I took the drug, I had many side effects and it put fear in me, so I stopped taking it for some time but now I have resumed and nothing will stop me because of the education you have just given* (Teacher).

*Yes, I took the drug once and that’s when I had problems. At that time I was a distributor. Since then I’ve not taken it, but now I’ll take it because, you (research team) said everybody is at risk in the community (volunteer distributor).*

*I take the drug as a community volunteer first and there is nothing that will stop me from taking the drugs. I have never defaulted and would not* (volunteer distributor).

*No, I have not taken the drugs since I started distributing it in the community, say 13 or 14 years now […] I was afraid of the side effects, I don’t want to become weak after taking it or to develop rashes and itches on my body. I may say that it was largely due to fear and the perception that the drug will make you weak after and sick. […] I will start taking it now that you told us that all of us were at risk of developing the disease* (volunteer distributor).

*Yes I took it for the first time last year […] I used to be afraid of side effects but now, nothing will stop me from taking it because when I took it last year, I didn’t experience any side effects* (volunteer distributor).

### The role of community-based volunteer distributors

Community members were divided in their views on the works of the distributors, while some were satisfied others were not. Various reasons were given for not being satisfied with the work of the distributors. It came to light that the distributors came into the community at any time of day without regards for the time suitable for the people. Distributors reportedly start the distribution of the drugs at their own convenience, which was often late morning after 8 am, by which time most people have left for their places of work. Others reported that, the distributors behave like health professionals who are in the communities to help ‘sick people’ and therefore make no effort to explain or convince people as to why they should take the drug. It also emerged that some of the distributors ask for favours from the people in the forms of cash, food and drinks. Some also, reportedly, smells of alcohol whilst distributing the drugs, thus offending people. These positions were captured in the following representative narratives:

*I am not happy with the volunteer […] he is not patient with people and sometimes even demand money or gift from us before giving the drugs. I think he should not be allowed to distribute drugs. His behaviour has put many people off that they have stopped taking the drug* (Male opinion leader).

*Also because the distributor is not from this community, he works only in the morning, meanwhile he does not come very early too […] he does not come in the evening, let alone on weekend* (female opinion leader).

*I don’t like the work of the distributor because he behaves like a doctor who is there to help us […] we are not well and he was sent to give us drugs and whether we take or not, it is our own problem […] I know I am not sick, so why should I take the medicine? Meanwhile we all live in this same community, so if he is not sick then what shows that I am sick with my family members? […] his actions have made a lot of people to stop taking the drug that I can tell you* (male opinion leader).

Being satisfied with the work of the distributors was based on a number of factors. Some people were satisfied with them based on personal relationships that they have with the distributors. Others were impressed with the work dedication of the volunteers who would follow up on persons who were absent when the distributors first visit their homes. These positions were represented in the following narratives:

*I have no problem with distributors; except that they have to understand the community in terms of our movements […] they are part of this community and must know when we are at home* (Female opinion leader).

*I do not have anything against the work of the volunteers but I don’t want them to come to my house with the drug because of my previous experience, where I suffered after taking the drugs* (Male opinion leader).

*I had no problem with the distributors because he is doing his work and he does it well […] if he comes and you are at home he will give you the drug and it is left to you to take it or refuse* (Female opinion leader).

### Drug taking fatigue

Drug taking fatigue was not reported as a reason for not taking the medication. Respondents maintained that, one cannot get tired of taking medicines once in a year and insisted that the issue was that some people could not understand why they should continue to take drugs when they are not sick, especially when they were told that the medicine is for lymphatic filariasis (elephantiasis and hydrocele). Some respondents maintain that they have heard some people complained about the continuous drugs taking even though they were not sick. Some also claimed that the drug was not doing anything for them, except causing them some discomforts like rashes and itching among others. Other respondents mentioned that they did not think that people were tired of ingesting the drug because it is taken just once in 12 months and that taking a drug once in a year cannot warrant fatigue. The following representative narratives captured respondents’ sentiments.

*I think it is not so much about people getting tired of the drugs because it is only once in a whole year […] I think it has to do with the side effects of the drug and the feeling that they were not sick. I am yet to hear anyone say he is tired of the drug* (Volunteer distributor).

*Oh, I don’t think so […] what I know is that many people, especially the youth, have stop taking the drugs because they don’t have the disease. Some of them don’t even react to the drugs but they have stopped and I think peer pressure is also playing a role* (Male opinion leader).

*I took the drugs for five times and stopped but it was not because I was tired of the drugs* per se*, but due to the reactions that I had […] it was very uncomfortable, it makes you sick (Female opinion leader).*

## Discussion

The success of any disease control and elimination effort requires not only the active participation of those it is designed for, but must also be compatible with local needs and understanding of perceived causes, transmission, treatment and prevention [15]. Findings show that residents were aware of the MDA intervention that has been going on for more than 12 years but the level of participation in terms of actual ingestion of the drugs varied significantly with very few taking the drugs consistently over the years and this might be at the centre of the emergence of the hotspot phenomenon.

Findings indicate that some people have either never taken the medicine or have stopped taking it for fear of side effects. It was reported from Haiti that the fear of side effects was the second highest reported reason for noncompliance [16]. It has also been reported that when patients see medication as ineffective or have experienced unwanted side effects, they are likely to stop taking the medication [17]. For MDA programmes, the chances of stopping could even be higher since most of the expected clienteles are not patients. Programme implementers must, therefore, be very open to discuss the side effects frankly with the people and assure them that most of the effects may not require medical interventions. The fact that every medicine is likely to activate some reactions in some individuals must be emphasized, and more importantly, the people must be told what to do in case of any adverse reaction after taking the drugs. The assurance that the health system will take care of those who experience adverse events must be emphasized and this also requires that the available health facilities are sensitized to respond appropriately to such cases. All these will help to build the confidence of people in the MDA programme, which may lead to compliance, especially ingestion of drugs to enhance the effectiveness of the programme to interrupt transmission as desired.

It was reported from Haiti that noncompliance was significantly associated with infection, suggesting continuous transmission of LF was linked to systematic noncompliance to MDA, especially ingestion of the programme drugs [16]. In our study, we found that some people have never taken the drugs at all, while others have stopped taking it because they think they were not susceptible to the infection. This has confirmed the assertion that patients who see themselves as susceptible to a disease condition were more likely to adhere to treatment [16–18]. In our study, people without obvious clinical signs and manifestations of the disease do not see why they should continue to take the medication and this need to be addressed. We believe that this could be addressed through rigorous community engagements and mobilization for MDA promotional activities including educational campaigns. This position is supported by what was reported from India, where an educational campaign delivered one month prior to the MDA had led to an increase in MDA compliance from 59.5% to 90.2% and 52.2% to 75% [18].

The finding that some people were not ingesting the drugs consistently purely based on personal dislike for medicines must be addressed by continuous interactions between the programme implementers and community members to stress the importance of every qualified person taking the medicine. It should be emphasised that there is a need for everybody to take the medicine appropriately in order to safeguard the future generations from the misery of the disease, especially its debilitating effects like elephantiasis and hydrocele. The fact that it takes few people to keep transmission going on in the community must be clearly made known to community members.

The health system related barriers concern the activities of community-based volunteer distributors and the supervision role of programme implementers/managers during MDA. It has been reported that health system related factors could affect adherence to medication or a treatment plan [17–20] and to ensure adherence to MDA, the district health system must play active roles in community mobilization efforts and perform active supervisions during MDA. They must visit communities during MDA to supervise the distributors to ensure compliance to protocols. This will help build community members’ confidence in the programme to enhance participation and therefore ingestion of the drugs. The practice whereby some communities do not have local residents as volunteer distributors must be rectified such that each community will be allowed to pick distributors from among themselves, this may be very vital for community mobilization and mop-up exercises during MDA to enhance participation.

The current practice where distributors are evaluated based on the leftover drugs, to indicate success and coverage, must be reviewed as it leaves room for unscrupulous distributors to tick or mark people who did not receive any drug as though they did and thereby reportedly throw away the drugs to cover up for their dishonesty. This finding must be taken very seriously because it was reported by community-based distributors themselves. It was not, therefore, a surprise that it was reported from a systematic review that adherence assessed using pill counts reported higher levels of adherence even when compared to those using self-report [17]. It may be good to do some random visits to selected homes to check if residents have taken the medicine. This will help to reduce the fraud significantly as perpetrators are likely to be found, and even if they were not found out, it will serve as a check to get them do the right thing since they will not know which homes will be randomly visited.

The issue of health facilities demanding for payment before treating those who reported with adverse events must be addressed as it was one of the main reasons why some people have stopped taking the drugs. Public health facility managers in MDA implementation areas must be sensitized to be ready to receive such cases and treat them for free or at worst, charge the cost to the programme.

Like any qualitative research, this study was not looking for principles that are true all the time and in all conditions (like laws of physics); rather, the goal was to understand specific circumstances, how and why things (in this case, the inability of MDA to interrupt transmission after 15 rounds as against the estimated/modelled 5–7 rounds) actually happen in a complex world. Simply put, findings reported here is situational and conditional, which may change overtime [14]. Also, findings should not be generalised to cover all ‘hotspots’ in Ghana, let alone all endemic areas experiencing the hotspot phenomenon, since qualitative research does not aim at generalizations but in-depth description of the views of the study population.

## Conclusion

In conclusion however, this qualitative study has gauged the actual sentiments and feelings of respondents regarding MDA in general and drug ingestion in particular and findings could be used to inform programme implementation to achieve the desired effect of interrupting LF transmission in hotspot communities. Findings could also inform the design and implementation of similar studies in hotspot communities to generate locally relevant data for the improvement of MDA programme implementation.

## Abbreviations

ADL: Adenolymphangitis
ALB: Albendazole
DEC: Diethylcarbamazine
EDCTP: Europeans and Developing countries Clinical Trial Programme
FWA: Federal Wide Assurance
GHS-ERC: Ghana Health Service-Ethical Review Committee
GPELF: Global programme to Eliminate Lymphatic Filariasis
IVM: Ivermectin
LF: Lymphatic Filariasis
MDA: Mass Drug Administration
MF: Microfilariae
NMIMR IRB: Noguchi Memorial Institute for Medical Research Institutional Review Board
